# Supplementary material for: No pump, no problem: evaluating passive eDNA sampling for marine biomonitoring of a nuisance macroalga
Source: PeerJ. 2025 Aug 25;13:e19939. doi: 10.7717/peerj.19939 (PMC12393076; doi:10.7717/peerj.19939)
Supplement: Supplemental Information 1 — Passive eDNA samplers (PEDS) were deployed at three atolls: Hōlanikū, or Kure Atoll, Kuaihelani, or Midway Island, and Manawai, or Pearl & Hermes Atoll. Chondria tumulosa benthic cover was estimated within an approximately 314 m2 area by two surveyors at each site. Exposure duration was 15 min. for all deployments of PEDs on stationary buoys, but varied when attached directly to SCUBA divers. The number of qPCR technical replicates with positive quantification cycle (Cq) values are listed for both biological samples per site. Posterior probabilities and credible intervals (CI) are listed for parameter estimates from site-occupancy modeling. The number of positive qPCR detection replicates per sample is listed. The method of PEDS deployment is also provided (active MCE vs. passive MCE, passive cotton vs. passive MCE on buoys, or passive cotton on SCUBA). [file peerj-13-19939-s001.docx]

| **Site** | **Location** | **Date** | **Depth (m)** | **% Cover** | **Exposure (min.)** | **Pos. PCRs** | **Presence *z* (CI)** | **Occupancy** *ψ* **(CI)** | ***θ*_11_ (CI)** | ***p*_11_ (CI)** | **Method** |
| --- | --- | --- | --- | --- | --- | --- | --- | --- | --- | --- | --- |
| H02 | Hōlanikū | 7/11/2023 | 1.8 | <1 | 15 | 4, 4 | 1 | 0.60  (0.36, 0.83) | 0.98  (0.85, 1) | 0.79  (0.66, 0.90) | Active MCE & Passive MCE |
| H04 | Hōlanikū | 7/11/2023 | 1.5 | <1 | 15 | 4, 5 | 1 | 0.60  (0.35, 0.84) | 0.98  (0.85, 1) | 0.75  (0.59, 0.89) | Active MCE & Passive MCE |
| H05 | Hōlanikū | 7/11/2023 | 1.5 | <1 | 15 | 4, 5 | 1 | 0.60  (0.36, 0.83) | 0.98  (0.85, 1) | 0.79  (0.66, 0.89) | Active MCE & Passive MCE |
| H09 | Hōlanikū | 7/12/2023 | 1.8 | 0 | 15 | 0, 0 | 0 | 0.59  (0.36, 0.82) | 0.97  (0.85, 1) | 0.82  (0.71, 0.91) | Active MCE & Passive MCE |
| H10 | Hōlanikū | 7/12/2023 | 0.9 | 0 | 15 | 0, 0 | 0 | 0.59  (0.36, 0.82) | 0.97  (0.85, 1) | 0.81  (0.70, 0.91) | Active MCE & Passive MCE |
| K01 | Kuaihelani | 2/13/2024 | 3.7 | 5 | 15 | 6, 6 | 1 | 0.61  (0.39, 0.81) | 0.96  (0.83, 1) | 0.92  (0.82, 0.97) | Active MCE & Passive MCE |
| K02 | Kuaihelani | 6/26/2024 | 3.7 | 1 | 15 | 3, 3 | 1 | 0.54  (0.33, 0.74) | 0.93  (0.78, 1) | 0.49  (0.35, 0.63) | Passive Cotton & MCE on Buoy |
| K05 | Kuaihelani | 2/13/2024 | 1.5 | 85 | 15 | 6, 6 | 1 | 0.83  (0.44, 1) | 0.93  (0.39, 1) | 0.98  (0.90, 1) | Active MCE & Passive MCE |
| K07 | Kuaihelani | 6/26/2024 | 2.7 | <1 | 15 | 3, 3 | 1 | 0.54  (0.33, 0.74) | 0.94  (0.78, 1) | 0.47  (0.32, 0.61) | Passive Cotton & MCE on Buoy |
| K10 | Kuaihelani | 6/26/2024 | 4.6 | 20 | 15 | 3, 3 | 1 | 0.64  (0.42, 0.84) | 0.94  (0.79, 1) | 0.61  (0.42, 0.81) | Passive Cotton & MCE on Buoy |
| K11 | Kuaihelani | 6/26/2024 | 2.7 | 1 | 15 | 0, 3 | 1 | 0.54  (0.33, 0.74) | 0.94  (0.78, 1) | 0.48  (0.34, 0.62) | Passive Cotton & MCE on Buoy |
| K12 | Kuaihelani | 2/13/2024 | 1.5 | 5 | 15 | 6, 6 | 1 | 0.61  (0.39, 0.81) | 0.96  (0.84, 1) | 0.91  (0.82, 0.96) | Active MCE & Passive MCE |
| K13 | Kuaihelani | 2/13/2024 | 2.7 | 80 | 15 | 6, 6 | 1 | 0.82  (0.44, 0.99) | 0.93  (0.43, 1) | 0.98  (0.90, 1) | Active MCE & Passive MCE |
| K14 | Kuaihelani | 6/27/2024 | 7.6 | 0 | 15 | 0, 0 | 0 | 0.54  (0.32, 0.74) | 0.93  (0.77, 1) | 0.48  (0.34, 0.63) | Passive Cotton & MCE on Buoy |
| K15 | Kuaihelani | 6/27/2024 | 1.8 | 0 | 15 | 0, 0 | 0 | 0.54  (0.32, 0.74) | 0.93  (0.77, 1) | 0.49  (0.34, 0.63) | Passive Cotton & MCE on Buoy |
| K16 | Kuaihelani | 6/27/2024 | 0.9 | 0 | 15 | 0, 0 | 0 | 0.54  (0.32, 0.74) | 0.93  (0.77, 1) | 0.49  (0.34, 0.64) | Passive Cotton & MCE on Buoy |
| K17 | Kuaihelani | 2/13/2024 | 1.1 | 20 | 15 | 6, 6 | 1 | 0.68  (0.46, 0.87) | 0.96  (0.83, 1) | 0.94  (0.87, 0.98) | Active MCE & Passive MCE |
| K20 | Kuaihelani | 6/27/2024 | 1.8 | <1 | 15 | 3, 3 | 1 | 0.54  (0.33, 0.74) | 0.93  (0.78, 1) | 0.48  (0.33, 0.62) | Passive Cotton & MCE on Buoy |
| K21 | Kuaihelani | 6/27/2024 | 4.6 | 0 | 15 | 0, 0 | 0 | 0.54  (0.32, 0.74) | 0.93  (0.77, 1) | 0.49  (0.34, 0.64) | Passive Cotton & MCE on Buoy |
| K22 | Kuaihelani | 6/27/2024 | 1.5 | 0 | 15 | 0, 0 | 0 | 0.53  (0.32, 0.74) | 0.93  (0.76, 1) | 0.51  (0.35, 0.66) | Passive Cotton & MCE on Buoy |
| K23 | Kuaihelani | 6/27/2024 | 0.9 | 0 | 15 | 0, 0 | 0 | 0.54  (0.32, 0.74) | 0.93  (0.77, 1) | 0.49  (0.34, 0.64) | Passive Cotton & MCE on Buoy |
| K24 | Kuaihelani | 6/27/2024 | 0.9 | 0 | 15 | 0, 0 | 0 | 0.54  (0.32, 0.74) | 0.93  (0.77, 1) | 0.48  (0.34, 0.63) | Passive Cotton & MCE on Buoy |
| PHR01 | Manawai | 9/23/2024 | 6.1 | 0 | 47 | 1, 2 | 0.08  (0, 1) | 0.50  (0.18, 0.78) | 0.38  (0.04, 0.91) | 0.97  (0.87, 1) | Passive cotton on SCUBA |
| PHR02 | Manawai | 9/23/2024 | 3.4 | 0 | 57 | 6, 0 | 0.17  (0, 1) | 0.50  (0.19, 0.78) | 0.39  (0.05, 0.90) | 0.97  (0.89, 1) | Passive cotton on SCUBA |
| PHR03 | Manawai | 9/26/2024 | 4.6 | 0 | 223 | 1, 3 | 0.07  (0, 1) | 0.46  (0.08, 0.86) | 0.43  (0, 1) | 0.95  (0.27, 1) | Passive cotton on SCUBA |
| PHR04 | Manawai | 9/26/2024 | 21.3 | 0 | 225 | 1, 0 | 0.07  (0, 1) | 0.46  (0.08, 0.86) | 0.43  (0, 1) | 0.94  (0.26, 1) | Passive cotton on SCUBA |
| PHR05 | Manawai | 9/27/2024 | 4.3 | <1 | 49 | 0, 0 | 1 | 0.51  (0.23, 0.78) | 0.51  (0.14, 0.91) | 0.96  (0.86, 1) | Passive cotton on SCUBA |
| PHR06 | Manawai | 9/27/2024 | 3.0 | 0 | 15 | 6, 3 | 0.28  (0, 1) | 0.52  (0.24, 0.79) | 0.55  (0.11, 0.93) | 0.91  (0.66, 1) | Passive cotton on SCUBA |
| PHR07 | Manawai | 9/24/2024 | 3.0 | 60 | 54 | 6, 5 | 1 | 0.77  (0.42, 0.98) | 0.59  (0.08, 1) | 0.96  (0.80, 1) | Passive cotton on SCUBA |
| PHR08 | Manawai | 9/24/2024 | 3.0 | 60 | 46 | 4, 6 | 1 | 0.77  (0.43, 0.98) | 0.61  (0.10, 1) | 0.96  (0.84, 1) | Passive cotton on SCUBA |
| PHR09 | Manawai | 9/27/2024 | 3.7 | <1 | 53 | 6, 6 | 1 | 0.51  (0.25, 0.77) | 0.62  (0.27, 0.92) | 0.95  (0.83, 1) | Passive cotton on SCUBA |
| PHR10 | Manawai | 9/25/2024 | 20.7 | 20 | 225 | 6, 6 | 1 | 0.62  (0.20, 0.93) | 0.67  (0, 1) | 0.95  (0.47, 1) | Passive cotton on SCUBA |
| PHR11 | Manawai | 7/18/2023 | 16.8 | 15 | 90 | 3, 6 | 1 | 0.60  (0.35, 0.83) | 0.64  (0.23, 0.95) | 0.97  (0.86, 1) | Passive cotton on SCUBA |
| PHR12 | Manawai | 7/18/2023 | 16.8 | 0 | 52 | 0, 5 | 0.19  (0, 1) | 0.51  (0.23, 0.77) | 0.53  (0.16, 0.91) | 0.96  (0.86, 1) | Passive cotton on SCUBA |

Note: Presence is the estimated probability that *C. tumulosa* eDNA is present at a site, given all modeled observations. Occupancy (*ψ*) is the inherent probability that a site is occupied by *C. tumulosa* eDNA. True capture (*θ*_11_) is the probability of a sample containing *C. tumulosa* eDNA from an occupied site. True detection (*p*_11_) is the probability of qPCR replicate detection from a sample containing *C. tumulosa* eDNA.
