# Supplementary material for: No pump, no problem: evaluating passive eDNA sampling for marine biomonitoring of a nuisance macroalga
Source: PeerJ. 2025 Aug 25;13:e19939. doi: 10.7717/peerj.19939 (PMC12393076; doi:10.7717/peerj.19939)
Supplement: Supplemental Information 2 — Results from an analysis of covariance (ANCOVA) testing for PCR inhibition by comparing Cq values and log-transformed DNA starting quantities (log10(StartingQuantity)) between synthetic DNA standards and standards spiked with eDNA matrix (“Template Type”). [file peerj-13-19939-s002.docx]

**Table S2. Linear model for testing of qPCR inhibition.** Results from an analysis of covariance (ANCOVA) testing for PCR inhibition by comparing C_q_ values and log-transformed DNA starting quantities (log10(StartingQuantity)) between synthetic DNA standards and standards spiked with eDNA matrix (“Template Type”).

| **Term** | **Degrees of freedom (df)** | **Sum of Squares (SS)** | **F-value** | **p-value** |
| --- | --- | --- | --- | --- |
| log10(Starting Quantity) | 1 | 735.40 | 579.05 | < 0.001 |
| Template Type | 1 | 3.71 | 2.92 | 0.10 |
| log10(Starting Quantity):Template Type | 1 | 4.32 | 3.40 | 0.07 |
| Residuals | 36 | 45.72 |  |  |
