# Supplementary material for: No pump, no problem: evaluating passive eDNA sampling for marine biomonitoring of a nuisance macroalga
Source: PeerJ. 2025 Aug 25;13:e19939. doi: 10.7717/peerj.19939 (PMC12393076; doi:10.7717/peerj.19939)
Supplement: Supplemental Information 3 — Posterior mean and 95% credible interval (CI) for model regression coefficients: probability of true capture (θ11), false-positive inference capture (θ10), true detection (p11), and false-positive test detection (p10). The probabilities of false-negatives at the field stage (1-θ11) and laboratory stage (1-p11) are the complements of θ11 and p11, respectively. [file peerj-13-19939-s003.docx]

**Table S3. Comparison of model parameter estimates from the previous actively-filtered assay (Nichols et al. 2025) and the membranes in passive environmental DNA samplers (PEDS).** Posterior mean and 95% credible interval (CI) for model regression coefficients: probability of true capture (*θ*_11_), false-positive inference capture (*θ*_10_), true detection (*p*_11_), and false-positive test detection (*p*_10_). The probabilities of false-negatives at the field stage (1-*θ*_11_) and laboratory stage (1-*p*_11_) are the complements of *θ*_11_ and *p*_11_, respectively.

|  | **True capture**  **(*θ*_11_)** | **False-negative capture**  **(1- *θ*_11_)** | **False-positive capture**  **(*θ*_10_)** | **True detection**  **(*p*_11_)** | **False-negative detection**  **(1- *p*_11_)** | **False-positive test detection**  **(*p*_10_)** |
| --- | --- | --- | --- | --- | --- | --- |
| Active  (Nichols et al. 2025) | 0.89 (0.70, 1.0) | 0.11 (0, 0.30) | 0.03 (0, 0.10) | 0.94 (0.80, 1.0) | 0.06 (0, 0.20) | 0.02 (0, 0.10) |
| PEDS | 0.90 (0.70, 0.98) | 0.10 (0.02, 0.30) | 0.07 (0.01, 0.21) | 0.76 (0.54, 0.91) | 0.24 (0.09, 0.46) | 0.04 (0.01, 0.11) |
