# Supplementary figures and images for: No pump, no problem: evaluating passive eDNA sampling for marine biomonitoring of a nuisance macroalga
Source: PeerJ. 2025 Aug 25;13:e19939. doi: 10.7717/peerj.19939 (PMC12393076; doi:10.7717/peerj.19939)

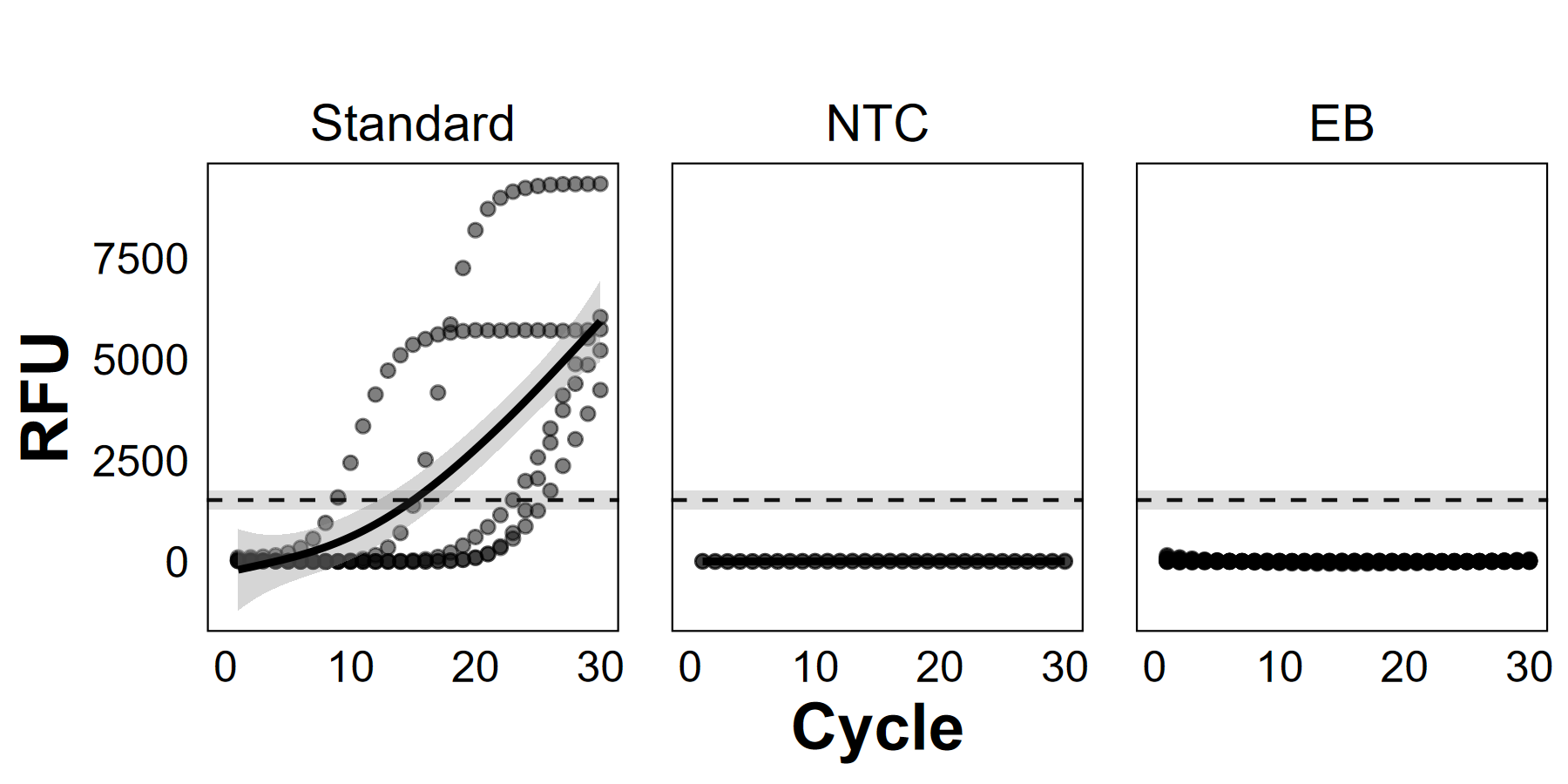

Supplement: Supplemental Information 4 — Quantitative polymerase chain reaction (qPCR) amplification curves from control samples: positive serial dilution standards from a stock solution of Chondria tumulosa tissue (“Standard”), qPCR no-template controls (“NTC”, n = 30), and field/equipment blanks (“EB”, n = 29). The mean fluorescence quantification threshold (±SE) is marked with a dashed grey line. [file peerj-13-19939-s004.png]

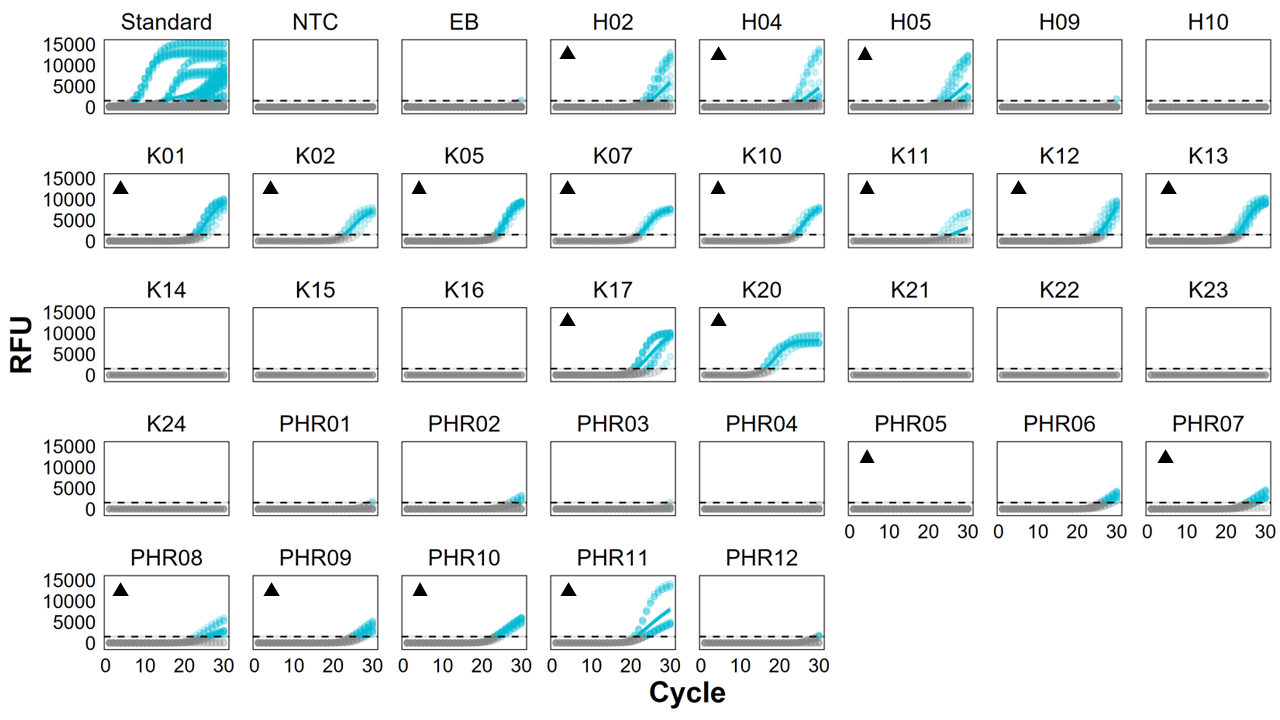

Supplement: Supplemental Information 5 — Sites were from Hōlanikū (“H”, or Kure Atoll), Kuaihelani (“K”, or Midway Island), and Manawai (“PHR”, or Pearl & Hermes Atoll). Amplification (relative fluorescence units, RFU) of Chondria tumulosa eDNA is marked with circles (representing each individual qPCR replicate) and a solid line generalized additive model smoother of triplicate PCR reactions among water samples from each site. Points and lines are colored blue if they exceed the average (±SE) threshold of detection. Control samples consisting of: positive C. tumulosa tissue extraction serial dilutions (“Standard”), qPCR no-template controls (“NTC”), and equipment blanks (“EB”). Sites with confirmed detections from direct visual surveys are marked with a (). [file peerj-13-19939-s005.png]

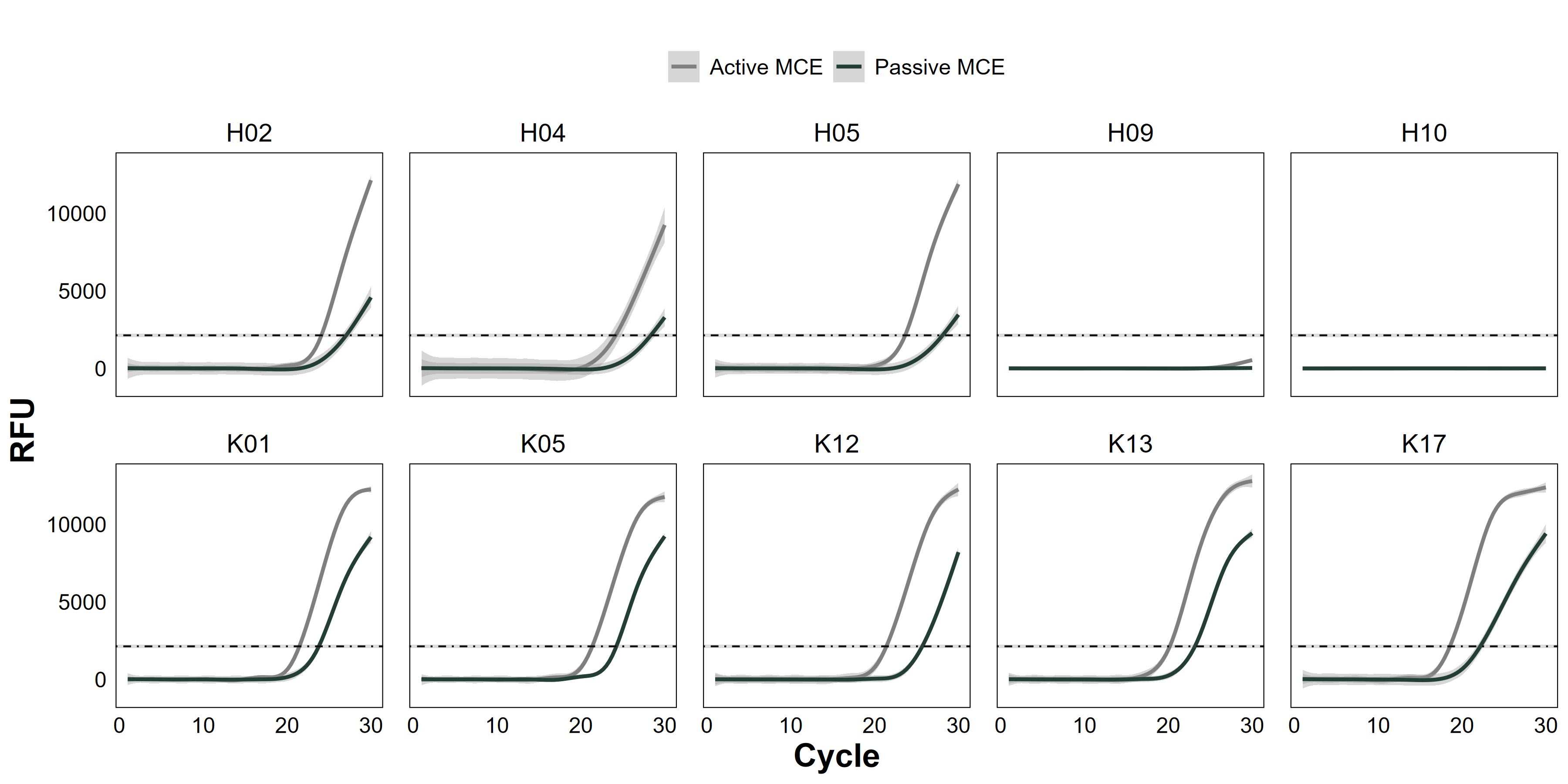

Supplement: Supplemental Information 6 — Comparison of environmental DNA (eDNA) quantitative polymerase chain reaction (qPCR) amplification curves from passive eDNA sampler (PEDS) deployments (“Passive MCE”, solid black line) and active filtration of 2-L water samples (“Active MCE”, solid grey line). Sites were from Hōlanikū (“H”, or Kure Atoll) or Kuaihelani (“K”, or Midway Island). Amplification (relative fluorescence units, RFU) of Chondria tumulosa eDNA is marked with a best-fit generalized additive model smoother of triplicate PCR reactions among water samples from each site. The mean fluorescence quantification threshold (±SE) is marked with a dashed grey line. [file peerj-13-19939-s006.png]

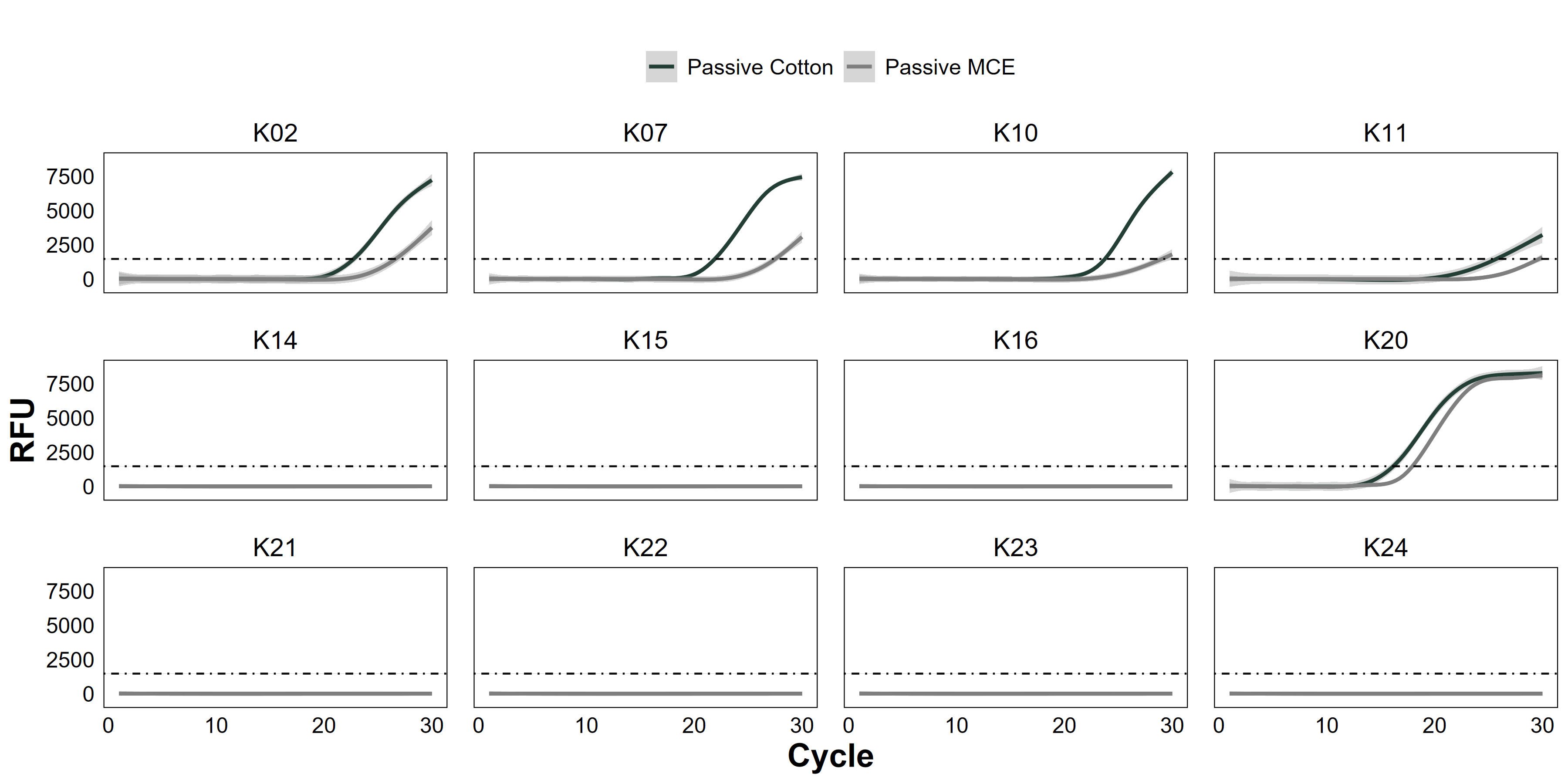

Supplement: Supplemental Information 7 — Comparison of environmental DNA (eDNA) quantitative polymerase chain reaction (qPCR) amplification curves from passive eDNA sampler (PEDS) deployments using cotton membranes (“Passive Cotton”, solid black line) or mixed cellulose ester analytical filters (“Passive MCE”, solid grey line). Sites were from Hōlanikū (“H”, or Kure Atoll) or Kuaihelani (“K”, or Midway Island). Amplification (relative fluorescence units, RFU) of Chondria tumulosa eDNA is marked with a best-fit generalized additive model smoother of triplicate PCR reactions among water samples from each site. [file peerj-13-19939-s007.png]
